# IL-6 Versus TNF-α as Predictors of Echocardiographic Cardiac Remodeling in Maintenance Hemodialysis Patients

**DOI:** 10.3390/medicina61091667

**Published:** 2025-09-14

**Authors:** Alexandru Florin Sircuța, Iulia Dana Grosu, Adalbert Schiller, Ligia Petrica, Viviana Ivan, Oana Schiller, Marcel Palamar, Monica-Nicoleta Mircea, Daniel Nișulescu, Ionuț Goleț, Flaviu Bob

**Affiliations:** 1 Department of Internal Medicine II—Nephrology University Clinic, “Victor Babes” University of Medicine and Pharmacy, Eftimie Murgu Sq. No. 2, 300041 Timișoara, Romania; alexandru.sircuta@umft.ro (A.F.S.);; 2 Centre for Molecular Research in Nephrology and Vascular Disease, Faculty of Medicine, “Victor Babes” University of Medicine and Pharmacy, Eftimie Murgu Sq. No. 2, 300041 Timișoara, Romania; 3 County Emergency Hospital, L. Rebreanu Street, Nr. 156, 300723 Timișoara, Romania; ivan.viviana@umft.ro; 4 Department of Internal Medicine II—Cardiology University Clinic, “Victor Babes” University of Medicine and Pharmacy, Eftimie Murgu Sq. No. 2, 300041 Timișoara, Romania; 5 B Braun Avitum Dialysis Centre, 300417 Timișoara, Romania; 6 Institute of Cardiovascular Diseases Timișoara, 13A Gheorghe Adam Street, 300310 Timisoara, Romania; 7 Research Center of the Institute of Cardiovascular Diseases Timisoara, 13A Gheorghe Adam Street, 300310 Timișoara, Romania; 8 Department of Management, Faculty of Economics and Business Administration, University of the West, 300115 Timișoara, Romania

**Keywords:** hemodialysis, Interleukin-6, Tumor Necrosis Factor-α, left ventricular hypertrophy, echocardiography, chronic inflammation, cardiac remodeling, right ventricular dilation

## Abstract

*Background and Objectives*: Cardiovascular disease remains the leading cause of mortality in end-stage renal disease, with systemic inflammation implicated in myocardial remodeling. We aimed to assess the associations between IL-6, TNF-α, and IL-1β and echocardiographic parameters of cardiac remodeling, including left ventricular mass (LVM), global longitudinal strain (GLS), interventricular septum (IVS), left ventricular end-diastolic diameter (LVEDD), and right ventricular diameter (RVD), in patients undergoing maintenance hemodialysis. This was a single-center retrospective observational study. *Materials and Methods*: In 58 maintenance hemodialysis patients (mean age 60.4 ± 11.7 years; 55% male), pre-dialysis serum cytokines (IL-6, TNF-α, IL-1β) and standard laboratory markers (C-reactive protein [CRP], albumin, hemoglobin) were measured. Echocardiography was performed under clinically stable conditions. Spearman correlations assessed relationships between cytokines and imaging parameters; multivariate linear regression identified independent predictors. *Results*: Median IL-6 was 7.36 pg/mL (interquartile range [IQR] 4.52–11.03), and median TNF-α was 9.35 pg/mL (IQR 7.9–12.57). IL-6 correlated positively with LVM (ρ = 0.63, *p* < 0.001), RVD (ρ = 0.53, *p* < 0.001), and CRP (ρ = 0.52, *p* < 0.001). In contrast, TNF-α inversely correlated with LVM (ρ = −0.36, *p* = 0.006). Multivariate regression showed IL-6 was independently predicted by LVM (*p* = 0.019) and RVD (*p* = 0.042), while TNF-α was predicted by age (*p* < 0.001), CRP (*p* = 0.038), and albumin (*p* = 0.012). *Conclusions*: In hemodialysis patients, IL-6 showed stronger associations with echocardiographic hypertrophy and dilation than TNF-α, supporting its role as a potential biomarker of subclinical cardiac remodeling.IL-6 showed stronger correlations with echocardiographic remodeling markers compared with TNF-α and may warrant further investigation as a potential biomarker in this setting.

## 1. Introduction

According to the International Society of Nephrology Global Kidney Health Atlas report, which pointed out that the Eastern and Central Europe region, which includes 20 countries including Albania, Poland, Romania, Hungary, Turkey, and others, has a median prevalence of treated kidney failure (KF) of 764 patients per million population (pmp), which is slightly higher than the global median of 759 pmp. In most of these countries, over 90% of people who require dialysis get hemodialysis (HD), which is the most frequently performed kind of treatment [1]. The same report brought out the rising incidence of cardiovascular disease (CVD) within this demographic, primarily attributed to an aging population and the significant prevalence of related risk factors, including hypertension, diabetes, and obesity in the region [1].

Cardiovascular disease is the primary cause of death in HD patients, accounting for over 50% of fatalities. Left ventricular hypertrophy (LVH), a response to increased cardiac workload, is prevalent in 60–75% of patients with ESKD receiving maintenance dialysis [2]. Moreover, sudden cardiac death (SCD) has emerged as the leading mode of mortality in this population, with recent evidence showing that nearly one quarter of cardiac arrest events occur in close temporal association with the hemodialysis procedure, clustering particularly after the long interdialytic interval and within the first post-dialysis hour [3]. This vulnerability reflects the combined impact of structural remodeling, myocardial fibrosis, and the chronic inflammatory state characteristic of HD patients. Apart from this, LVH, an adaptive response to increased cardiac work, is an independent risk factor for cardiovascular events and mortality in HD patients. This vulnerability is due to the complex pathophysiology of LV remodeling and dysfunction, including hemodynamic factors like hypertension and arterial stiffness, and uremia-related factors like anemia and mineral and bone metabolism disorder [2]. Hemodialysis significantly impacts the heart, causing a decrease in atrial and ventricular volumes due to ultrafiltration and intravascular depletion [4].

However, functional impairment is observed in the left atrium (LA) and ventricle (LV). Speckle-tracking echocardiography shows reduced LA reservoir and conduit strain, suggesting atrial mechanics alteration due to myocardial ischemia or hemodynamic shifts [4]. Globally diminished longitudinal strain (GLS) of the left ventricle decreases post dialysis, indicating transient myocardial dysfunction [4]. Monitoring GLS is crucial for identifying at-risk patients and implementing strategies to lower cardiac risks during dialysis [4].

Cardiovascular complications and echocardiographic changes are largely caused by chronic inflammation in hemodialysis patients, which is brought about by elevated levels of interleukin-6 (IL-6) and tumor necrosis factor-alpha (TNF-α). This chronic inflammation contributes not only to cardiovascular morbidity but also to an increased risk of mortality in this patient population. Effective management of inflammation through targeted therapies may help improve overall outcomes for hemodialysis patients. In patients with end-stage renal disease (ESRD), these pro-inflammatory cytokines diligently aid in cardiac remodeling, left ventricular hypertrophy, and heart failure alongside being significant indicators of systemic inflammation [5,6]. Consequently documented by echocardiographic findings, their pathogenic effects come along with endothelial dysfunction, arterial stiffness, and the activation of inflammatory and neurohormonal pathways. These factors all together build up the workload of the myocardium and impair both systolic and diastolic cardiac function [7,8].

We aimed to assess the associations between key inflammatory biomarkers—IL-6, TNF-α, and IL-1β—and echocardiographic indicators of cardiac remodeling, including LVM, GLS, IVS, LVEDD, and RVD, in a retrospective cohort of 58 patients undergoing maintenance hemodialysis. Our primary hypothesis was that IL-6 would demonstrate stronger associations with structural and functional cardiac alterations compared to TNF-α. A secondary objective was to evaluate whether IL-1β shows specific links with chamber dilatation parameters, thereby providing complementary insights into the pathways of cardiac remodeling in this high-risk population.

## 2. Materials and Methods

We conducted a retrospective cohort study to assess the impact of inflammatory markers and echocardiographic findings, as well as the association between these two types of markers in patients undergoing hemodialysis.

### 2.1. Study Design and Population

This was a retrospective cohort study conducted at the Avitum B. Braun Centre in Timişoara, Romania. Out of 138 patients undergoing maintenance hemodialysis in the center, 58 fulfilled the eligibility criteria and were consecutively enrolled during the study period. The 58 research subjects (≥18 years old) were all diagnosed with stage 5D chronic kidney disease (CKD), receiving maintenance hemodialysis using high-flux dialyzers three times a week for at least four hours per session.

Inclusion required ≥3 months on hemodialysis, age 18–75 years, stable clinical condition with regular attendance at hemodialysis sessions, and ability to obtain adequate echocardiographic images. Patients with significant noncompliance to hemodialysis (missed or skipped sessions, irregular attendance, or intolerance) were not included.

Exclusion criteria were represented by age > 75 years old, active infection within 30 days, active malignancy, recent (<6 months) myocardial infarction, EF < 30%, or inability to obtain adequate echocardiographic images, and chronic treatment with immunosuppressive or anti-inflammatory agents, since such therapies could significantly bias inflammatory biomarker levels. Medication records were reviewed for all patients, and none of the included subjects were receiving chronic immunosuppressive or anti-inflammatory therapy. Occasional short-term use of nonsteroidal anti-inflammatory drugs (NSAIDs) for intercurrent conditions could not be fully excluded if not reported by the patient at the time of enrollment.

All participants provided informed consent prior to study inclusion.

All procedures were authorized by the Ethics Committee of VICTOR BABES UNIVERSITY OF MEDICINE AND PHARMACY, TIMISOARA, ROMANIA (protocol code 33/30 June 2021), and were implemented in conformity with the Declaration of Helsinki and the institutional research committee’s ethical standards. The Dialysis Center’s Ethics Committee gave authorization for the current research. Written informed consent was requested from eligible patients before any study procedures were carried out.

### 2.2. Clinical and Laboratory Assessments

Baseline demographics (age, sex) and dialysis vintage (years on hemodialysis) were recorded. Height and weight were measured to calculate body mass index (BMI). Dry weight (post-dialysis) was recorded in kilograms. Before dialysis, fasting blood samples were taken from the arteriovenous fistula or central venous catheter after the longest break between dialysis sessions (samples were collected on Monday for patients who have dialysis on Monday, Wednesday, and Friday, and on Tuesday for those who have dialysis on Tuesday, Thursday, and Saturday). All blood samples were collected immediately prior to the hemodialysis session (predialysis), at the time of the patient’s scheduled dialysis shift (morning, noon, or evening). Cytokines such as IL-6 and TNF-α reflect chronic inflammatory status and are not expected to show significant short-term diurnal variability; therefore, predialysis sampling was considered appropriate for standardization. The samples were centrifuged at 1000× *g* for 10 min after being kept at 4 °C for about an hour. Serum portions were made after centrifugation and kept at −80 °C until additional examination.

Samples to measure particular biomarkers were gathered in line with each biomarker’s manual instructions.

The analysis included a complete blood count: platelet count (PLT, ×10^3^/µL), hemoglobin (g/dL); serum chemistry: albumin (g/dL), creatinine (mg/dL), urea (mg/dL), calcium (mg/dL), phosphorus (mg/dL), sodium (mEq/L), potassium (mmol/L), bicarbonate (mmol/L), and C-reactive protein (CRP, mg/dL). After this, we determined inflammatory biomarkers (measured by ELISA kits): interleukin-6 (IL-6, pg/mL), tumor necrosis factor-α (TNF-α, pg/mL), and interleukin-1β (IL-1β, pg/mL). Serum IL-6 concentrations were measured by electrochemiluminescence immunoassay (ECLIA) on frozen serum samples (minimum 1 mL, stored at −20 °C for up to 3 months, transported on dry ice; stability: 1 day at 2–8 °C, 3 months at −20 °C). TNF-α concentrations were determined by chemiluminescence immunoassay on frozen serum samples (minimum 1 mL, stability up to 6 months at −20 °C, transported on dry ice). All assays were performed in a single accredited laboratory to minimize variability, with routine internal quality controls performed according to the manufacturer’s instructions. Using an ELISA kit from Elabscience (Houston, TX, USA) with catalog number E-EL-H0149 and product size 96T/48T/24T/96T*5, interleukin 1-beta (IL-1β) was measured. The kit has a detection range of 7.81–500 pg/mL and a sensitivity of 4.69 pg/mL.

Transthoracic echocardiography was performed mid-dialysis by a single blinded cardiologist using a ESAOTE MyLabX8 Platform (ESAOTE, Genova, Italy) ultrasound systems equipped with a 1–5 MHz transducer. Standard parasternal long-axis, apical four-chamber, and two-chamber views were acquired.

The cardiac evaluation (Pulse Doppler, M-mode continuous, and two-dimensional) was carried out over the 2nd and the third hours of dialysis, respectively. The measurements were accomplished by a single cardiologist with the same echocardiography equipment to avoid bias. Echocardiography was carried out in compliance with the guidelines set forth by the European Association of Cardiovascular Imaging (EACVI). Left atrial diameter (LA), interventricular septum thickness (IVS), global longitudinal strain (GLS), left ventricular mass (LVM), E/A ratio, left ventricular end-diastolic diameter (LVEDD), end-systolic diameter (LVESD), and right ventricular basal diameter (RV) were all evaluated. Aortic atheromatosis, endomyocardial calcifications, aortic and mitral valve calcifications, and fibrosis were among the structural anomalies that were meticulously evaluated. All measurements represent the average of three consecutive beats. Intra-observer coefficients of variation for LVEDD, LVESD, IVS, and LVM were <5%.

All baseline data were analyzed retrospectively as part of an interim evaluation of the included participants.

### 2.3. Statistical Analysis

IBM SPSS Statistics for Windows, Version 30.0.0.0 (IBM Corp., Armonk, NY, USA), was used to conduct the statistical analyses. Continuous variables with normal distribution (as determined by the Shapiro–Wilk) are reported as mean ± SD; non-Gaussian variables as median (IQR). Categorical variables as number (percentage). Spearman’s rank correlation assessed associations between IL-6, TNF-α, IL-1β, and echocardiographic parameters. To further evaluate these relationships, univariate and multivariate linear regression models were constructed. Multivariate models were built using a backward stepwise selection procedure, with all candidate covariates entered initially; non-significant variables were excluded iteratively, and only significant predictors were retained in the final models. These analyses were designed to evaluate the independent associations of IL-6 and TNF-α with echocardiographic remodeling parameters, rather than to predict cytokine levels themselves, and not merely to dichotomize patients by the presence or absence of LVH. A two-tailed *p* < 0.05 was considered statistically significant.

## 3. Results

### 3.1. Patient Characteristics

Table 1 shows clinical and laboratory variables for all 58 patients.

The starting point parameters of the 58 participants in this research are shown in Table 1. The cohort had a slight male predominance (55%), a median dialysis vintage of 6.4 years (SD: 4.89), and an average age of 60.4 years (SD: 11.74). With a mean BMI of 29.17 kg/m^2^ (SD: 6.43) and a mean serum albumin of 4.07 g/dL (SD: 0.39), the nutritional status was largely maintained. The hemoglobin values (mean 10.83 g/dL, SD: 1.26) showed proper management of anemia. The electrolyte profiles were in line with what would be expected for a dialysis population: bicarbonate was 22.19 mmol/L (SD: 2.27), potassium was 5.42 mmol/L (SD: 0.64), phosphorus was 5.60 mg/dL (SD: 1.62), and calcium was 8.52 mg/dL (SD: 0.46). Uremic toxin levels were elevated, with a mean serum creatinine of 8.52 mg/dL and urea of 122.46 mg/dL, indicating a significant uremic burden. There was significant variation in the platelet count, which averaged 235.26 × 10^3^/µL (SD: 102.60).

### 3.2. Echocardiographic Findings

A summary of the cardiac parameters for each of the 58 participants is shown in Table 2.

The cohort’s left atrial diameter, which had a mean of 41.87 mm (SD: 4.70), was significantly larger. In 91.4% of patients, there was a high prevalence of aortic atheromatosis. Myocardial and valve calcifications were common: aortic valve calcifications occurred in 67.2% of cases, mitral valve calcifications in 77.6%, and endomyocardial calcifications in 70.7% of cases. Furthermore, both the aortic and mitral valves showed valvular fibrosis in 77.6% of the patients.

The mean left ventricular end-diastolic diameter (LVEDD) was 53.83 mm (SD: 6.21), and the end-systolic diameter (LVESD) was 38.64 mm (SD: 6.80), with echocardiographic measures showing structural remodeling. The left ventricular mass was high (mean 267.43 g, SD: 83.77), and the interventricular septal thickness reached 12.89 mm (SD: 1.65), suggesting concentric hypertrophy in a significant fraction of the group. Diastolic dysfunction was frequently noticed, as evidenced by a lower mean E/A ratio of 0.71 (SD: 0.34). At 49.29% (SD: 7.98), the mean ejection fraction showed a slight decline. A sensitive measure of subclinical systolic dysfunction, global longitudinal strain (GLS), has resulted in a mean of 14.68% (SD: 2.75). The median right ventricular diameter was 28 mm (IQR: 26–29), which may indicate early right ventricular involvement in some situations.

### 3.3. Inflammatory Markers

The table below displays the principal markers of inflammation illustrated by this investigation.

Table 3 illustrates the levels of key inflammatory biomarkers. With a median value of 7.36 pg/mL (IQR: 4.52–11.03), interleukin-6 (IL-6) levels were moderately increased, indicating low-grade systemic inflammation. Interleukin-1β (IL-1β) exhibited consistently high levels throughout the cohort, with a median of 44.44 pg/mL (IQR: 42.92–47.43), but tumor necrosis factor-alpha (TNF-α) levels were more tightly clustered around the median, with a value of 9.35 pg/mL (IQR: 7.90–12.57). Reference ranges are not provided because values are assay-dependent and not standardized for the hemodialysis population

The majority of the participants exhibited mild chronic inflammation, as indicated by the mean value of C-reactive protein (CRP), a general indicator of inflammation, of 1.09 mg/dL (SD: 1.73). In aggregate, these results support a long-standing pro-inflammatory profile in the hemodialysis population, with IL-6 showing up as the most dynamically changeable cytokine and possibly the most clinically relevant one for cardiac alterations.

### 3.4. Correlations Analyses Between Cytokines and Echocardiography

#### 3.4.1. Spearman’s Rank Correlations

Spearman’s rank correlations are shown in Table 4.

The strongest positive correlation was between IL-6 and LV mass (ρ = 0.630; *p* < 0.001), indicating that patients with higher IL-6 had significantly greater LV hypertrophy. TNF-α exhibited an inverse relationship with LV mass (ρ = −0.375; *p* = 0.006), suggesting a divergent inflammatory profile. IL-1β correlated with LVEDD (ρ = 0.410; *p* = 0.004) but not with LV mass. Other IL-1β correlations with echocardiographic parameters were not significant.

#### 3.4.2. Multivariate Regression Analyses

The regression results are presented in Table 5, Table 6, Table 7, Table 8 and Table 9.

Model A (IL-6, all predictors) was non-significant (*p* model = 0.831), with a negative adjusted R^2^ (−0.147), indicating overfitting when too many variables were entered simultaneously. No individual predictor reached statistical significance.

In the stepwise regression analysis, IL-6 was independently predicted by LV mass (*p* = 0.019) and RV (*p* = 0.042). Notably, although RV showed only a borderline correlation with IL-6 in bivariate analysis (ρ = 0.260; *p* = 0.053), it emerged as an independent predictor after multivariate adjustment, highlighting the importance of controlling for confounding covariates.

The stepwise regression model explained 38.7% of the variance in IL-6 (R^2^ = 0.387, adjusted R^2^ = 0.346, *p*_model = 0.015). Both LV mass (β = 0.548; t = 2.534; *p* = 0.019) and RV (β = 0.151; t = 2.063; *p* = 0.042) remained significant predictors. Each 1 g increase in LV mass corresponded to an average rise of 2.53 pg/mL in IL-6, while each 1 mm increase in RV corresponded to an increase of 0.61 pg/mL.

In the stepwise regression analysis, TNF-α was independently associated with age (*p* < 0.001), CRP (*p* = 0.038), and albumin (*p* = 0.012). All three predictors contributed significantly to explaining variability in TNF-α levels in this cohort.

The model explained 51.3% of the variance in TNF-α (R^2^ = 0.513, adjusted R^2^ = 0.286, *p*_model < 0.001). Older age (β = 0.606; *p* < 0.001), higher CRP (β = 0.418; *p* = 0.038), and higher albumin (β = 0.425; *p* = 0.012) were all independent predictors, suggesting that TNF-α reflects both systemic inflammation and nutritional status in this dialysis population.

## 4. Discussion

### 4.1. Key Findings

Our study adds to the existing literature by showing that IL-6 is more strongly linked to echocardiographic structural and functional remodeling in maintenance hemodialysis (HD) patients than TNF-α or IL-1β. Greater left ventricular mass (LVM) and right ventricular diameter (RVD) were independently linked to IL-6, indicating that it serves as a general indicator of structural stress. TNF-α, on the other hand, had a weaker and less specific association with cardiac remodeling; it correlated inversely with LVM but was otherwise only related to systemic factors like age, CRP, and albumin. Although IL-1β was not associated with LVM or GLS, it was somewhat associated with LVEDD, which might indicate that it plays a part in early ventricular dilatation as opposed to hypertrophy.

### 4.2. Comparison with Previous Studies

First and foremost, according to a meta-analysis performed by Chen et al., hemodialysis patients’ increased IL-6 levels were independently linked to both cardiovascular and all-cause mortality, confirming IL-6’s function as a prognostic indicator in this group [9].

In CKD patients, elevated levels of IL-6 and TNF-α have been linked to left ventricular hypertrophy, arterial stiffness, and increased cardiovascular mortality [10]. IL-6 was identified as a more accurate indicator of echocardiographic cardiac remodeling compared to TNF-α or IL-1β, according to this study. Genetic evidence from dialysis patients suggested that IL-6 directly contributes to cardiac remodeling. Specifically, HD patients with IL-6 −174G/C polymorphism (GC or CC genotypes) exhibit a significantly higher prevalence of LVH and increased LVM than those with the GG genotype, as reported by Losito et al. [11]. This indicates that chronic IL-6-mediated inflammation may directly cause cardiac structural changes in people receiving dialysis.

Our findings align with broader evidence on inflammation and cardiac remodeling in CKD. For instance, a recent study in non-dialysis CKD patients [12] also reported significant associations between IL-6 and structural cardiac changes, emphasizing its role as a driver of maladaptive remodeling. Unlike our hemodialysis cohort, their population was not exposed to dialysis-related hemodynamic stressors, yet the inflammatory link remained consistent. This suggests that IL-6 may represent a common pathway across different CKD stages.

Similarly, the recent multicenter analysis [13], highlighted the prognostic relevance of IL-6 and TNF-α for adverse cardiovascular outcomes in CKD. In that research [13], IL-6 emerged as a stronger predictor than TNF-α, mirroring our results in HD patients. However, while their study [13] focused on outcomes such as mortality and hospitalization, ours demonstrates echocardiographic structural remodeling as an intermediate phenotype, adding mechanistic insight.

Taken together, these studies [12,13] reinforce the robustness of IL-6 as a biomarker across CKD populations, while our work specifically extends this evidence to the hemodialysis setting, where cardiac remodeling is accelerated and clinically relevant.

Kamińska et al. [14], in a cohort of CKD patients not yet on dialysis, demonstrated a strong association between elevated IL-6 and coronary artery calcification as well as cardiovascular mortality, whereas TNF-α showed no such relationship. Even though their study evaluated cardiovascular risk based on coronary artery calcification (CAC) instead of direct echocardiographic modifications, both sets of findings underscore IL-6 as a more clinically significant biomarker in renal disease compared to TNF-α.While LVH emerged as a relevant correlate, our analytic strategy was not limited to comparing patients with and without LVH. Instead, we sought to evaluate IL-6 and TNF-α in relation to multiple echocardiographic remodeling parameters, in order to capture a broader perspective of cytokine–cardiac interactions. This approach should be regarded as hypothesis-generating and complementary to subgroup analyses that might be pursued in larger cohorts.

Our research identified IL-6 as a crucial cytokine linked to ventricular remodeling in HD patients in line with recent data compiled by Cruz Junho et al. [15]. Like our findings, their study highlighted the critical role that chronic inflammation, particularly mediated by IL-6, plays in the development of myocardial fibrosis, LVH, and compromised cardiac function in patients with CKD and dialysis, as well as its prognostic value for cardiovascular mortality [15]. Nonetheless, Cruz Junho et al. [15] highlighted the role of uremic toxins in CKD-associated cardiomyopathy, while our study was focused on the role of cytokines in predicting cardiac remodeling. However, IL-6 is positioned at the core of the inflammatory–cardiac connection in both viewpoints. This is further supported by extensive research. IL-6 is one of the cytokines that are raised early and persistently in CKD, according to Kaesler et al. [16]. The CRIC trial [17] demonstrated that in patients with CKD, elevated levels of IL-6 and high-sensitivity C-reactive protein (hs-CRP) were strongly related to LVH and systolic dysfunction [17]. Other research has linked worsening heart dysfunction to endotoxemia, which is characterized by elevated levels of endotoxin, IL-6, and CRP [18]. On top of that, another analysis revealed that cytokines like TNF-α and IL-10 are linked to vascular remodeling and inflammation, especially in younger CKD patients [19]. These observations are in contrast with our insights, which disclosed that TNF-α indicated only mild, nonspecific associations with systemic inflammatory factors such as CRP or serum albumin, while IL-6 was strongly associated with echocardiographic measurements of structural heart abnormalities.

Furthermore, higher IL-6 levels were linked to increased RVD, suggesting a connection between systemic inflammation and remodeling of the right heart. This finding agrees with the results of Karur et al. [20], who found that patients who were switched from conventional to nocturnal hemodialysis, a treatment known for better managing fluid and inflammation, showed a significant reduction in RVD. The FHN trials [21] showed that more frequent hemodialysis decreased both left ventricular mass and right ventricular end-diastolic volume, highlighting the impact of improved volume control on structural remodeling, which is complemented by our findings regarding ventricular remodeling. Moreover, a significant decrease in right ventricular end-diastolic volume (RVEDV) was also noted, indicating that, with the correct hemodynamic care, right heart dilatation might partially reverse [21].

### 4.3. Mechanistic Insights

In addition to this, the FHN data [21] support a parallel mechanism of remodeling driven by volume overload, whereas our study concentrated on inflammatory biomarkers like IL-6. This suggests that structural cardiac changes in hemodialysis patients are likely multifactorial, with both hemodynamic and inflammatory contributors.

In this context, the function of TNF-α deserves a comprehensive investigation. TNF-α, contrary to common belief, revealed weaker, more widespread relationships, primarily with systemic factors such as serum albumin, age, and CRP suggesting it reflects a general inflammatory state rather than specific heart remodeling in HD patients. Our findings align with human trials that have not consistently shown improved cardiac outcomes from TNF-α inhibition, despite its link to animal models of heart disease.

Therefore, instead of affecting cardiac structure, TNF-α in these HD patients may signify systemic inflammation, aligning with findings from other studies that corroborate this hypothesis [22,23].

Chronic inflammation driven by TNF-α promotes myocardial fibrosis, ventricular dilation, and progressive dysfunction in preclinical models, but this effect is more difficult to isolate in human trials due to compensatory mechanisms and comorbidities [22,24]. For example, Sun et al. found that mice lacking TNF-α developed less myocardial injury and hypertrophy after aortic banding compared to wild-type animals [24]. Likewise, in a rat model of volume overload, Matsumura et al. showed that inhibiting TNF-α with Etanercept reduced ventricular dilation and collagen loss [25]. These findings suggest a biologically plausible mechanism by which TNF-α could worsen structural cardiac damage.

But translating this into clinical benefit has proven difficult. Trials like ATTACH [26] and RENEWAL [27], which tested TNF-α inhibitors in heart failure patients, failed to show consistent improvements. Notably, both Infliximab and Etanercept failed in these randomized trials for heart failure, with high-dose Infliximab being associated with clinical worsening in the ATTACH study [27]. Disappointing results may stem from the complex, dual role of TNF-α via TNFR1 (inflammation/apoptosis) and TNFR2 (repair/survival), and the multifactorial nature of heart failure [22]. Global TNF-α inhibition may unintentionally suppress the protective TNFR2-mediated pathways, which could explain the disappointing outcomes in clinical trials [27,28]. TNF-α expression by immune cells, cardiomyocytes, and fibroblasts creates localized inflammation, complicating therapy as systemic blockade might not effectively modulate tissue-specific signaling [22]. The authors suggest a lack of success could also be attributed to limited understanding of chronic TNF-α behavior in humans [22]. As a whole, these observations point out that TNF-α, despite its molecular activity in preclinical models, fails to serve as a dependable biomarker for cardiac remodeling within the distinct inflammatory context of dialysis. In our investigation, TNF-α indicated diffuse inflammation instead of specific cardiac damage, highlighting the discrepancy between the theoretical and clinical outcomes.

Other findings from our study group solidify this interpretation. TNF-α exhibited a positive correlation with serum albumin levels, even when inside normal limits. This is different from what happens in systemic lupus erythematosus and other inflammatory diseases, where TNF-α levels go up as albumin levels go down [29]. Due to this difference, TNF-α may indicate a different inflammatory pattern in relation to dialysis, one that may be attributed to dietary and metabolic factors instead of overt protein-energy waste.

Remarkably, Supriyadi et al. [30] reported no significant link between TNF-α and albumin in a comparable group, but this could be attributed to their cohort’s lower albumin levels and higher incidence of HCV infections. The current study’s patients maintained their nutritional status, which may have affected the observed TNF-α dynamics.IL-1β exhibited a modest but significant correlation, aligning with LVEDD instead of LVM or GLS. This indicates a possible involvement in early ventricular dilation rather than concentric hypertrophy. In experimental chronic kidney disease (CKD), IL-1β facilitates atrial fibrosis and increases vulnerability to atrial fibrillation, effects that are mitigated by IL-1β blockade [31].

The research by Hung et al. [25], which showed that treating HD patients with IL-1 receptor antagonist (IL-1ra) resulted in a dramatic decrease in the blood levels of IL-6 and CRP without compromising hemodynamic stability or dialysis effectiveness supporting IL-1β as an upstream contributor to systemic inflammation. This supports previous findings in hypertensive patients, where LVEDD independently predicted cardiac events despite preserved systolic function [32]. Even after controlling for confounding variables, elevated LVEDD has been found to be an independent predictor of all-cause mortality in HD patients [33].

Apart from this, another study that included the HD population revealed that inflammation is closely linked to adipose and protein nutritional status [34]. TNF-α was a survival predictor in smokers, while IL-6 and IL-1β resulted in just general inflammatory markers [34]. Targeting specific inflammatory pathways could form a holistic patient management strategy.

### 4.4. Clinical Implications

Our findings reveal that IL-6 may function as a more useful indicator of cardiac remodeling compared to TNF-α or IL-1β. IL-6 was consistently linked to both LVM and RVD, which may help to identify patients that are at increased risk and need more frequent echocardiographic monitoring. This may be especially useful in dialysis centers without regular access to advanced imaging modalities like speckle-tracking echocardiography. Simultaneously, IL-6 ought to be considered a marker and not absolute validation of causality, due to the observational design of our study. Before it can be used in practice, a few problems need to be fixed. These include standardizing the assay, setting out clinically relevant cut-offs, and then analyzing its cost-effectiveness to improve standard risk stratification.

Additional justification for thinking of IL-6 as a therapeutic target comes from recently developed interventional research. The RESCUE phase 2 trial showed that Ziltivekimab, an anti–IL-6 monoclonal antibody, significantly lowered hs-CRP in patients with CKD [35]. More recently, a randomized phase 2b trial evaluating Clazakizumab in HD patients demonstrated profound reductions in hs-CRP (over 85%) without an increased risk of infections [36].

Major improvements in GLS, LV mass, and LA volume were reported in a recent study that looked at cardiac remodeling following kidney transplantation, especially in those with baseline strain impairment [37]. In contrast, our cohort of HD patients showed no signs of reverse remodeling.

GLS is becoming more widely acknowledged as a sensitive indicator of subclinical systolic dysfunction in current echocardiography guidelines [38]. Moreover, the 2021 ESC Heart Failure Guidelines recommend intervention when GLS declines by ≥12%, even if EF is preserved [39].

### 4.5. Limitations

There are several limitations of this research. The findings may not be as broadly applicable due to the single-center design and rather small sample size. Low-grade systemic inflammation, which is common in the hemodialysis population, could not be totally ruled out as a confounding factor, even though evident infections were eliminated. Furthermore, even though echocardiographic measurements are standardized, they still depend on the operator’s expertise. Given that reference ranges for TNF-α and IL-6 in hemodialysis patients are inconsistent and differ considerably between assays, we regarded the values found in our group as the most clinically meaningful benchmark. Blood samples were taken before dialysis based on the patient’s scheduled shift (morning, noon, or evening), however cytokines like TNF-α and IL-6 show chronic inflammation and are not significantly affected by short-term circadian fluctuations. Therefore, even though we admit that the sampling hour is not consistent, it is anticipated that this will have little effect on the outcomes.

## 5. Conclusions

Our research concluded that IL-6 is the most accurate inflammatory marker correlated with echocardiographic indicators of cardiac remodeling in HD patients especially when it refers to elevated left ventricular mass, impaired global longitudinal strain, and septal thickness. Whereas TNF-α obviously causes cardiac changes in animals, it is not a valid echocardiographic predictor in dialysis patients, according to our data. In the particular context of CKD, TNF-α does not indicate the path to cardiac remodeling, albeit it may still have supportive functions through inflammation.

Despite being mechanistically linked to cardiovascular disease, TNF-α and IL-1β showed weaker and less reliable correlations. According to these results, IL-6 has the potential to be a clinical target because, in contrast to TNF-α or IL-1β, it may indicate both inflammation and active structural alterations in the heart. These findings highlight the need for more research on IL-6-directed treatments as a means of lowering cardiovascular burden in this high-risk group.

## Figures and Tables

**Table 1:** General Parameters–Mean ± SD (n = 58).

Parameter	Mean	SD
Age (years)	60.40	11.74
Dialysis duration (years)	6.43	4.89
Dry weight (kg)	83.33	19.33
Body Mass Index (BMI, kg/m^2^)	29.17	6.43
Albumin (g/dL)	4.07	0.39
Platelet count (PLT, ×10^3^/µL)	235.26	102.60
Hemoglobin (g/dL)	10.83	1.26
Creatinine (mg/dL)	8.52	2.04
Urea (mg/dL)	122.46	26.99
Phosphorus (mg/dL)	5.60	1.62
Calcium (mg/dL)	8.52	0.46
Sodium (mEq/L)	138.51	3.10
Potassium (mmol/L)	5.42	0.64
Bicarbonate (mmol/L)	22.19	2.27

BMI—Body Mass Index; PLT—Platelet count; SD—Standard deviation.

**Table 2:** Cardiovascular & Valve Parameters—Descriptive.

Parameter	Value (Mean ± SD or n/%)
Left atrial diameter (LA, mm)	41.87 ± 4.70
Right ventricular diameter (RV, mm)	28 (26–29) *
Aortic atheromatosis N (%)	53/91.4%
Endomyocardial calcifications N (%)	41/70.7%
Aortic valve calcifications N (%)	39/67.2%
Aortic valve fibrosis N (%)	45/77.6%
Mitral valve calcifications N (%)	45/77.6%
Mitral valve fibrosis N (%)	45/77.6%
Left ventricular end-diastolic diameter (LVEDD, mm)	53.83 ± 6.21
Left ventricular end-systolic diameter (LVESD, mm)	38.64 ± 6.80
Interventricular septum thickness (IVS, mm)	12.89 ± 1.65
Left ventricular mass (LVM, g)	267.43 ± 83.77
E/A ratio	0.71 ± 0.34
Ejection fraction (EF, %)	49.29 ± 7.98
Global longitudinal strain (GLS, %)	14.68 ± 2.75
Endomyocardial calcifications (present)	41/70.7%

* Median (IQR) for RV (non-Gaussian); LA—Left atrial diameter; RV—Right ventricular diameter; LVEDD—Left ventricular end-diastolic diameter; LVESD—Left ventricular end-systolic diameter; IVS—Interventricular septum; LVM—Left ventricular mass; EF—Ejection fraction; GLS—Global longitudinal strain; E/A—Early-to-late diastolic flow ratio.

**Table 3:** Inflammation parameters analysis.

Parameter	Median (IQR)
IL-6 (pg/mL)	7.36 (4.52–11.03)
TNF-α (pg/mL)	9.35 (7.90–12.57)
IL-1β (pg/mL)	44.439 (42.924–47.432)
CRP (mg/dL)	1.091 (0.249–1.210)

IL-6—Interleukin-6; TNF-α—Tumor necrosis factor-alpha; IL-1β—Interleukin-1 beta; CRP—C-reactive protein; IQR—Interquartile range.

**Table 4:** Spearman Correlations between Cytokines and Echocardiographic Parameters.

Cytokine	Echo Parameter	ρ (Rho)	*p*-Value
**IL-6**	LV mass	0.630	<0.001
	RV	0.260	0.053
	IVS	0.107	0.676 (NS)
	E/A ratio	0.074	0.744 (NS)
	EF	0.105	0.670 (NS)
	GLS	0.315	0.244 (NS)
**TNF-α**	LV mass	−0.375	0.006
	RV	−0.147	0.637 (NS)
	IVS	0.090	0.651 (NS)
	E/A ratio	−0.032	0.858 (NS)
	EF	0.124	0.455 (NS)
	GLS	0.217	0.469 (NS)
**IL-1β**	LVEDD	0.410	0.004
(NS)	Other echo params	—	>0.050

NS = not significant; LV = left ventricle; RV = right ventricle; IVS = interventricular septum; EF = ejection fraction; GLS = global longitudinal strain; LVEDD = left ventricular end-diastolic diameter; IL = interleukin; TNF = tumor necrosis factor.

**Table 5:** Regression Model A: IL-6 with All Predictors (n = 45 usable cases).

Metric	Value
R	0.494
R^2^	0.244
Adjusted R^2^	−0.147
Std. Error of Estimate	24.355
F (df 1 = 15, df 2 = 29)	0.624
*p*-value for overall model (Sig. F)	0.831 (NS)
Number of predictors initially tested	15
Number of cases (df 2 = 29)	45
Metric	Value

NS = not significant; R^2^—Coefficient of determination; SE—Standard error; df—Degrees of freedom. No individual predictor reached *p* < 0.05 in this “all-in” model.

**Table 6:** Regression Model B: IL-6 (Stepwise, Retained Predictors).

Predictor	B	SE	β	t	*p*	95% CI
LV mass (LVM, g)	2.534	1.347	0.548	2.534	0.019	0.507 to 4.561
RV (RV, mm)	0.605	0.293	0.151	2.063	0.042	0.022 to 1.188

LVM—Left ventricular mass; RV—Right ventricular diameter; CI—Confidence interval; SE—Standard error.

**Table 7:** Regression Model B: IL-6 with Retained Predictors; Model statistics (n = 45).

Metric	Value
R	0.622
R^2^	0.387
Adjusted R^2^	0.346
Std. Error	21.295
F (2, 42)	8.569
*p* (model)	0.015 *

* *p* < 0.05. Each 1 g increase in LV mass corresponds to a 2.53 pg/mL rise in IL 6; each 1 mm increase in RV corresponds to a 0.61 pg/mL rise. R^2^—Coefficient of determination.

**Table 8:** Regression Model C: TNF-α (Stepwise, Retained Predictors).

Predictor	B (Unstandardized)	Std. Error	β (Standardized)	t-Value	*p*-Value	95% CI for B
Age (years)	0.168	0.042	0.606	3.962	< 0.001 (**)	0.082 to 0.254
CRP (mg/dL)	0.797	0.367	0.418	2.173	0.038 (*)	0.052 to 1.542
Albumin (g/dL)	3.415	1.279	0.425	2.670	0.012 (*)	0.802 to 6.028

CRP—C-reactive protein; CI—Confidence interval; SE—Standard error; (*) *p* < 0.05; *p* < 0.001 (**).

**Table 9:** Regression Model C: TNF-α with Retained Predictors (Model statistics (n = 58).

Metric	Value
R	0.716
R^2^	0.513
Adjusted R^2^	0.286
Std. Error of Estimate	2.844
F (df 1 = 3, df 2 = 44)	4.602
*p* (model)	<0.001 (*)

* *p* < 0.05. Each 1 g increase in LV mass corresponds to a 2.53 pg/mL rise in IL 6; each 1 mm increase in RV corresponds to a 0.61 pg/mL rise. R^2^—Coefficient of determination.

## Data Availability

The data presented in this study are available on request from the corresponding author.

## Abbreviations

The following abbreviations are used in this manuscript:

ASE	American Society of Echocardiography
BMI	Body Mass Index
CAC	Coronary Artery Calcification
CKD	Chronic Kidney Disease
CRP	C-Reactive Protein
CRIC	Chronic Renal Insufficiency Cohort
CVD	Cardiovascular Disease
EACVI	European Association of Cardiovascular Imaging
EF	Ejection Fraction
ELISA	Enzyme-Linked Immunosorbent Assay
ESC	European Society of Cardiology
ESKD	End-Stage Kidney Disease
ESRD	End-Stage Renal Disease
FHN	Frequent Hemodialysis Network
GLS	Global Longitudinal Strain
HD	Hemodialysis
IL-1β	Interleukin-1 beta
IL-6	Interleukin-6
IQR	Interquartile Range
IVS	Interventricular Septum
LA	Left Atrium
LV	Left Ventricle
LVEDD	Left Ventricular End-Diastolic Diameter
LVESD	Left Ventricular End-Systolic Diameter
LVH	Left Ventricular Hypertrophy
LVM	Left Ventricular Mass
RV	Right Ventricle
RVD	Right Ventricular Diameter
SD	Standard Deviation
SPSS	Statistical Package for the Social Sciences
TNF-α	Tumor Necrosis Factor-alpha
